# Efficacy of *Mycobacterium vaccae* and vitamin D combination therapy in initial smear-negative pulmonary tuberculosis: a prospective study

**DOI:** 10.3389/fmed.2026.1778652

**Published:** 2026-04-22

**Authors:** Renkui Gu, Luyao Wang

**Affiliations:** Department of Infectious Disease, Aksu Third People's Hospital, Aksu, China

**Keywords:** cytokines, immunotherapy, *Mycobacterium vaccae*, pulmonary tuberculosis, T cell subsets, vitamin D

## Abstract

**Purpose:**

To evaluate the efficacy of adjunctive *Mycobacterium vaccae* (*M. vaccae*) and vitamin D in patients with initial smear-negative pulmonary tuberculosis (PTB) and to explore their effects on immune responses.

**Methods:**

In this prospective cohort study, 391 patients with initial smear-negative pulmonary tuberculosis were consecutively enrolled and followed for 4 months. Patients were assigned to one of three treatment groups: standard anti-tuberculosis therapy (control), standard therapy plus *M. vaccae* vaccination, and standard therapy combined with *M. vaccae* and vitamin D. Cytokine levels [interferon-γ (IFN-γ), interleukin-4 (IL-4), IL-17, IL-10], T cell subset ratios [Th1/Th2, Th17/regulatory T cells (Treg)], and clinical outcomes were prospectively assessed.

**Results:**

The combined therapy group showed significant increases in IFN-γ and Th1/Th2 ratio, and decreases in IL-4, IL-17, IL-10, and an increase in the Th17/Treg ratio, indicating a shift toward Th1-dominant immunity. Clinically, lesion absorption (54.33%) and cavity closure (71.13%) rates were highest in the combined group, with a total effective rate of 89.76%, surpassing the control (76.12%) and *M. vaccae* (79.23%) groups (*p* < 0.05).

**Conclusion:**

Adjunctive therapy with *M. vaccae* and vitamin D enhances immune function and improves clinical outcomes in initial smear-negative PTB, suggesting a synergistic immunotherapeutic effect.

## 1 Introduction

Tuberculosis, a chronic infectious disease caused by *Mycobacterium tuberculosis* (MTB), remains a leading cause of death from infectious diseases worldwide. Despite significant advancements in diagnosis, treatment, and vaccine development in recent years, the global tuberculosis epidemic continues to pose a formidable challenge (1). According to the World Health Organization’s *Global Tuberculosis Report 2025*, there were an estimated 10.6 million new cases and 1.3 million deaths in 2023, with 410,000 cases of multidrug-resistant/rifampicin-resistant tuberculosis (MDR/RR-TB) (1–3). The pathogenesis of tuberculosis is intricate and not yet fully understood. However, it is well-established that the disease’s progression is closely linked to the host’s immune status. Immunotherapy has emerged as a crucial component in the treatment of tuberculosis, aiming to modulate the immune response to expedite sputum conversion, enhance treatment success rates, and reduce the duration of therapy (4, 5).

The standard chemotherapy regimen for tuberculosis typically spans 6 months, which many patients find difficult to adhere to, leading to disease relapse or the development of drug resistance. *Mycobacterium vaccae* (*M. vaccae*), a heat-killed and purified freeze-dried vaccine, is a specific immunomodulator recommended by the World Health Organization for tuberculosis immunotherapy (6). Recent research has highlighted the complex interplay between the development of pulmonary tuberculosis (PTB) and the dynamic imbalance of CD4 + T cell subsets, particularly the ratio of helper T cells 1 (Th1) to Th2 (Th1/Th2) and the ratio of Th17 to regulatory T cells (Treg) (7–9). The injectable *M. vaccae* vaccine, known as “*M. vaccae*,” is an immunomodulatory agent recommended by the World Health Organization in its tuberculosis research and development strategy, demonstrating significant clinical efficacy in the treatment of newly diagnosed smear-negative PTB, with a low incidence of adverse reactions (10). Its combination with other treatments has been shown to markedly enhance immune function and therapeutic outcomes in PTB patients (11).

Clinical data indicate that over 50% of PTB patients have significantly lower levels of serum 25-hydroxyvitamin D3 (vitamin D) compared to healthy individuals. Serum vitamin D levels are positively correlated with peripheral blood CD4 + T cells and the CD4+/CD8 + T cell ratio, suggesting that vitamin D can modulate immune responses in PTB patients, enhancing T-cell bactericidal activity against MTB (12). Consequently, vitamin D deficiency is associated with an increased risk of MTB infection, and vitamin D supplementation has been shown to be effective in the prevention and treatment of PTB (13).

In a review of the diagnostic and therapeutic progress in smear-negative PTB patients, Meca et al. (14) emphasized the importance of initiating treatment from the outset to prevent further transmission. However, the implementation of such treatment is often hindered by the non-specific symptoms of smear-negative PTB patients, making radiological examination the primary diagnostic method. This leads to challenges in diagnosis and widespread issues with patient compliance (15). Therefore, exploring a treatment regimen that is both efficacious and short in duration, with high patient adherence, is a direct and effective measure to reduce the incidence of smear-negative tuberculosis. *M. vaccae*, known for its immune therapeutic and preventive effects, is an efficacious immunomodulator for tuberculosis (16). It can enhance the cellular immune function of PTB patients, accelerate the absorption of lesions and closure of cavities, reduce tissue pathological damage, and improve the clinical cure rate of PTB. *M. vaccae* is primarily administered through intramuscular injection, while its oral formulation (V7) is still in the clinical trial phase. Yang et al. (17) reported that *M. vaccae* treatment effectively promotes sputum conversion and lesion absorption in multidrug-resistant tuberculosis, reducing the recurrence rate.

Based on these findings, it is hypothesized that the combination of *M. vaccae* and vitamin D may significantly enhance the immune function and clinical efficacy in PTB patients. However, there is a scarcity of research on the combined adjuvant therapy of PTB with these two agents, and further studies are needed to confirm their potential synergistic effects.

## 2 Methods

### 2.1 Study participants

This study was conducted as a prospective cohort study. Patients newly diagnosed with smear-negative pulmonary tuberculosis at Aksu District Third People’s Hospital between March 2022 and September 2024 were consecutively recruited and followed for 4 months according to a predefined study protocol. According to previous studies, an expected difference in treatment effectiveness of 12–15% between groups was assumed (18). Based on a one-way ANOVA design (power = 80%, *α* = 0.05), the minimum required sample size was estimated to be 108 participants per group. Considering a 10% dropout rate, at least 120 participants per group were required. Patients were assigned to one of three treatment groups: routine anti-tuberculosis treatment (Control Group), routine anti-tuberculosis plus *M. vaccae* vaccine treatment (*M. vaccae* Group), and routine anti-tuberculosis plus *M. vaccae* vaccine plus vitamin D adjunctive treatment (Combined Group), and followed for 4 months according to predefined procedures. Enrollment was based on all eligible cases within the study period, and patients who withdrew or were lost to follow-up before completing the 4-month treatment regimen were excluded. Ultimately, 134 cases in the Control Group, 130 in the *M. vaccae* Group, and 127 in the Combined Group, totaling 391 PTB cases, were included in the analysis. Additionally, 400 healthy individuals who underwent physical examinations during the same period were selected as a healthy control group.

Inclusion criteria:

① Newly diagnosed, smear-negative pulmonary tuberculosis patients aged 18 to 65 years who have not previously received the *M. vaccae* vaccine.
② Diagnosis meets the criteria for smear-negative pulmonary tuberculosis:
③ Three consecutive negative sputum smear test results;
④ Chest radiograph showing active pulmonary lesions;
⑤ Exclusion of other non-tuberculous pulmonary diseases;
⑥ Meeting 1 to 2 of the above criteria, combined with medical history and clinical symptoms, determined by the diagnostic team.

Exclusion criteria:

① Contraindications for *M. vaccae* vaccine or fixed-dose combination (FDC) anti-tuberculosis drugs;
② Severe cardiac, hepatic, renal, or joint disorders;
③ No history of psychiatric, neurological diseases, epilepsy, immune dysregulation disorders, or extrapulmonary tuberculosis;
④ Peripheral white blood cells < 4.0 × 10^9/L before treatment, abnormal liver and kidney function, fasting blood glucose exceeding normal values;
⑤ Use of other immunomodulatory agents within the past 3 months.

The study protocol was approved by the Medical Ethics Committee of Aksu District Third People’s Hospital (Approval No.: 201418), and written informed consent was obtained from all participants. This study was registered at ClinicalTrials.gov under registration number NCT04975738, registered on July 23, 2021.

### 2.2 Data extraction and interventions

Data were collected prospectively from enrolled patients using standardized case report forms.

*Control group*: standard anti-tuberculosis therapy, including isoniazid, rifampicin, pyrazinamide, and ethambutol.

*M. vaccae group*: standard therapy plus *M. vaccae* vaccination. The vaccine (0.1 mL per dose, strain: *M. vaccae* [NCTC 11659], Beijing Weiqi Biological Pharmaceutical Co., Ltd., Beijing, China) was administered intradermally once weekly during the intensive phase (0–2 months) and once every 2 weeks during the consolidation phase (2–4 months).

*Combined group*: *M. vaccae* therapy as above with adjunctive vitamin D supplementation (cholecalciferol, 800 IU/day, DSM Nutritional Products, Basel, Switzerland), with doses adjusted according to recorded serum calcium levels.

Laboratory indicators were collected at baseline and after 4 months of treatment. Fasting venous blood was used for mononuclear cell isolation and flow cytometry analysis of Th1 (IFN-γ + CD4+), Th2 (IL-4 + CD4+), Th17 (IL-17 + CD4+), and Treg (CD4 + CD25 + Foxp3+) cells. Intracellular staining was performed following standard protocols using fluorochrome-conjugated monoclonal antibodies (IFN-γ, clone B27; IL-4, clone 8D4-8; IL-17, clone eBio64DEC17; Foxp3, clone PCH101; all from eBioscience, San Diego, CA, USA), and analyzed on a BD FACSCalibur flow cytometer (BD Biosciences, San Jose, CA, USA).

Serum cytokine levels (IFN-γ, IL-4, IL-17, IL-10) were measured via enzyme-linked immunosorbent assay (ELISA) according to the manufacturer’s instructions (eBioscience, San Diego, CA, USA).

Data quality was ensured through standardized procedures and independent verification by two trained researchers, with discrepancies resolved by consensus.

### 2.3 Outcome measures

The primary outcome of this study was clinical efficacy after 4 months of treatment, evaluated by the total effective rate. Secondary outcomes included radiological outcomes, specifically pulmonary cavity evaluation and lesion absorption, as well as immunological outcomes, including changes in cytokine levels and T cell subset ratios. Clinical efficacy was classified as follows: cured, defined as complete resolution of clinical symptoms and radiographic lesions; markedly improved, defined as significant improvement in symptoms and at least 50% lesion absorption on imaging; improved, defined as moderate improvement in symptoms and 30–50% lesion absorption; ineffective, defined as no improvement or worsening of symptoms and/or lesions. The total effective rate was calculated as the sum of cured, markedly improved, and improved cases (19, 20). Pulmonary cavity evaluation included closure, reduction, unchanged, and enlargement. Lesion absorption was assessed as absorption ≥1/2, significant absorption 1/3–1/2, unchanged <1/3, or deterioration.

All measurements were performed according to standardized protocols. All clinical outcomes and radiological evaluations were independently assessed by two experienced physicians who were blinded to the treatment allocation. In cases of disagreement, a third senior physician adjudicated the final evaluation to ensure objectivity and consistency. This study was not fully double-blinded due to the nature of the interventions, but the use of independent, blinded outcome assessors minimized potential bias.

### 2.4 Statistical analysis

All analyses were conducted retrospectively using SPSS 21.0 (IBM, Armonk, NY, USA). Continuous variables with a normal distribution are presented as x̄ ± s. Between-group comparisons were performed using independent-sample *t*-tests for two groups or one-way ANOVA for three or more groups, with LSD *post-hoc* tests for pairwise comparisons. Within-group changes were analyzed using paired t-tests. Categorical variables are expressed as *n* (%) and compared using the chi-square test. Ordinal data among multiple groups were analyzed with the Kruskal-Wallis H test. A two-sided *p*-value < 0.05 was considered statistically significant.

## 3 Result

### 3.1 Demographic and clinical characteristics

Baseline demographic and clinical characteristics of patients with initial smear-negative pulmonary tuberculosis were comparable among the Control, *M. vaccae*, and Combined Therapy groups (Table 1). Mean BMI values were similar across groups, within the normal range. Smoking and alcohol consumption rates showed minor variation, with the highest rates observed in the Combined Therapy group (smoking 24.48%, drinking 14.24%). Pleural effusion was most common in the Combined Therapy group (18.99%), and this group also had the highest number of pulmonary cavities (105).

**Table 1:** Baseline characteristics of patients with initial smear-negative pulmonary tuberculosis.

Characteristic	Control group (*n* = 134)	*M. vaccae* group (*n* = 130)	Combined therapy group (*n* = 127)	*χ* ^2^ */F*	*p*
Sex, *n* (%)					
Male	71 (52.99)	65 (49.62)	75 (59.06)	2.199	0.333
Female	63 (47.01)	65 (50.38)	52 (40.94)		
Age (years), mean ± SD	41.03 ± 2.81	42.03 ± 3.52	41.60 ± 2.87	0.630	0.533
BMI (kg/m^2^), mean ± SD	21.14 ± 1.57	21.57 ± 1.34	21.38 ± 1.08	1.214	0.298
Smoking, *n* (%)	28 (21.03)	25 (19.38)	31 (24.48)	1.063	0.588
Drinking, *n* (%)	12 (9.16)	15 (11.62)	18 (14.24)	1.743	0.418
Pleural effusion, *n* (%)	19 (14.35)	18 (13.95)	24 (18.99)	1.558	0.459

Data are presented as mean ± SD for continuous variables and as *n* (%) for categorical variables. BMI, body mass index.

### 3.2 Cytokine levels and Th1/Th2 and Th17/Treg ratios

Compared with healthy controls, patients with PTB exhibited a pronounced imbalance in immune cytokine profiles (Table 2). Specifically, Th1-associated immunity was suppressed, whereas Th2- and Th17-related cytokines were elevated, resulting in significantly lower Th1/Th2 and Th17/Treg ratios.

**Table 2:** Detection of Th1/Th2 and Th17/Treg system and related cytokine levels in different groups.

Biomarker	Healthy (*n* = 400)	PTB (*n* = 391)	*t*	*p*
IFN-γ (pg/mL)	32.19 ± 5.45	9.61 ± 2.21	58.83	< 0.001
IL-4 (pg/mL)	22.94 ± 3.76	50.41 ± 5.20	67.95	< 0.001
Th1/Th2	2.46 ± 0.60	1.67 ± 0.43	20.80	< 0.001
IL-17 (pg/mL)	10.27 ± 1.98	35.35 ± 4.01	86.67	< 0.001
IL-10 (pg/mL)	22.49 ± 2.53	70.43 ± 8.41	82.84	< 0.001
Th17/Treg	0.62 ± 0.13	0.23 ± 0.15	51.98	< 0.001

Data are presented as mean ± SD. Comparisons between the Healthy Control and PTB groups were performed using independent samples *t*-tests. PTB, pulmonary tuberculosis; IFN-γ, interferon-gamma; IL, interleukin; Th, T helper cell; Treg, regulatory T cell.

### 3.3 Changes in Th1/Th2 cytokine levels post-treatment

Following 4 months of treatment, all groups exhibited significant modulation of Th1/Th2 cytokine profiles (Table 3). IFN-γ levels increased while IL-4 levels decreased, resulting in a pronounced elevation of the Th1/Th2 ratio, indicative of a shift toward a Th1-dominant immune response. The Combined Therapy Group demonstrated the most marked improvements compared with the Control and *M. vaccae* Groups, with between-group differences reaching statistical significance (*p* < 0.001).

**Table 3:** Changes in Th1/Th2 cytokines before and after 4-month treatment across different groups.

Biomarker	Timepoint	Control	*M. vaccae*	Combined	Time effect (*P*)	Group effect (*P*)	Time × group interaction (*P*)
IFN-γ (pg/mL)	Baseline	9.28 ± 2.50	9.81 ± 2.15	9.74 ± 2.08	< 0.001	< 0.001	< 0.001
	Month 4	12.47 ± 1.86	14.29 ± 3.58^*^	16.26 ± 2.80^#†^			
IL-4 (pg/mL)	Baseline	50.54 ± 5.20	50.34 ± 5.19	50.35 ± 5.22	< 0.001	< 0.001	< 0.001
	Month 4	39.02 ± 2.29	37.36 ± 1.91^*^	36.67 ± 2.14^#†^			
Th1/Th2	Baseline	1.64 ± 0.45	1.68 ± 0.34	1.72 ± 0.41	< 0.001	< 0.001	0.001
	Month 4	2.21 ± 0.50	2.32 ± 0.38^*^	2.45 ± 0.52^#†^			

Data are presented as mean ± SD. Analyzed using two-way repeated-measures ANOVA to assess time, group, and interaction effects. IFN-γ, interferon-gamma; IL-4, interleukin-4; Th, T helper cell; SD, standard deviation; *M. vaccae*, *Mycobacterium vaccae*. ^*^p < 0.05 vs Control; ^#^*p* < 0.05 vs Control for Combined group; ^†^*p* < 0.05 vs *M. vaccae* for Combined group.

### 3.4 Changes in Th17/Treg cytokine levels post-treatment

After 4 months of treatment, all groups exhibited significant modulation of Th17/Treg cytokine profiles (Table 4). IL-17 and IL-10 levels decreased, accompanied by a pronounced increase in the Th17/Treg ratio, indicating a shift toward a more balanced immune response. The Combined Therapy Group showed the most marked improvements compared with the Control and *M. vaccae* Groups, with between-group differences reaching statistical significance (*p* < 0.001).

**Table 4:** Changes in Th17/Treg cytokines before and after 4-month treatment in three groups.

Biomarker	Timepoint	Control	*M. vaccae*	Combined therapy	Time (*P*)	Group (*P*)	Time × group (*P*)
IL-17 (pg/mL)	Baseline	35.31 ± 4.30	34.80 ± 4.48	36.00 ± 3.88	< 0.001	< 0.001	< 0.001
	Month 4	29.54 ± 3.41	28.57 ± 2.30^*^	27.24 ± 3.72^#†^			
IL-10 (pg/mL)	Baseline	71.53 ± 8.39	69.82 ± 7.09	70.00 ± 8.43	< 0.001	< 0.001	< 0.001
	Month 4	50.38 ± 4.36	43.72 ± 4.20^*^	35.60 ± 4.08^#†^			
Th17/Treg	Baseline	0.14 ± 0.46	0.14 ± 0.49	0.12 ± 0.44	< 0.001	< 0.001	< 0.001
	Month 4	0.26 ± 0.28	0.34 ± 0.31^*^	0.45 ± 0.34^#†^			

Data are presented as mean ± SD. Analyzed using two-way repeated-measures ANOVA to assess time, group, and interaction effects. IL-17, interleukin-17; IL-10, interleukin-10; Th17, T helper 17 cell; Treg, regulatory T cell; SD, standard deviation; M. vaceae, Mycobacterium vaceae. ^*^p < 0.05 vs Control; ^#^*p* < 0.05 vs Control for Combined group; ^†^*p* < 0.05 vs *M. vaccae* for Combined group.

### 3.5 Pulmonary lesion and cavity improvement post-treatment

After 4 months of treatment, all groups demonstrated notable improvements in pulmonary lesions and cavity conditions (Tables 5, 6). The Combined Therapy Group exhibited the most pronounced lesion absorption and cavity closure, followed by the *M. vaccae* Group, with the Control Group showing the least improvement. Between-group comparisons revealed statistically significant differences in cavity closure rates (*p* = 0.034), supporting the superior efficacy of the combined therapeutic approach.

**Table 5:** Pulmonary lesion improvement after 4 months of treatment in patients with initial smear-negative pulmonary tuberculosis.

Group	n	Absorption, *n* (%)	Significant absorption, *n* (%)	Unchanged, *n* (%)	Worsening, *n* (%)	Total improved, *n* (%)
Control	134	42 (31.34)	63 (47.01)	22 (16.42)	7 (5.22)	105 (78.36)
*M. vaccae*	130	53 (40.77)	56 (43.08)	19 (14.62)	2 (1.54)	109 (83.85)
Combined	127	69 (54.33)^#^	48 (37.79)^#^	10 (7.87)^#^	0 (0.00)^#^	117 (92.13)^#^

Overall differences among groups were assessed using the Kruskal–Wallis test (*p* = 0.034), followed by post hoc pairwise comparisons using the Mann–Whitney U test with Bonferroni correction, which showed a significant difference between the Control and Combined groups (*p* = 0.010). ^#^*p* < 0.05 vs Control for Combined group.

**Table 6:** Pulmonary cavity improvement after 4 months of treatment in patients with initial smear-negative pulmonary tuberculosis.

Group	*n*	Closure, *n* (%)	Reduction, *n* (%)	Unchanged, *n* (%)	Enlargement, *n* (%)	Total improved, *n* (%)
Control	103	28 (27.18)	59 (57.28)	13 (12.62)	2 (1.94)	87 (84.47)
*M. vaccae*	99	57 (57.58)^*^	37 (37.37)^*^	5 (5.05)^*^	0 (0.00)^*^	94 (94.95)^*^
Combined	97	69 (71.13)^#^	28 (28.87)^#^	0 (0.00)^#^	0 (0.00)^#^	97 (100)^#^

Overall differences among groups were assessed using the Kruskal–Wallis test (*p* = 0.034), followed by post hoc pairwise comparisons using the Mann–Whitney U test with Bonferroni correction, which showed significant differences between the Control and *M. vaccae* groups (*p* = 0.034) and between the Control and Combined groups (*p* < 0.001), whereas no significant difference was found between the *M. vaccae* and Combined groups (*p* = 0.201). ^*^p < 0.05 vs Control; ^#^*p* < 0.05 vs Control for Combined group.

### 3.6 Efficacy analysis post-treatment

**Figure 1:**
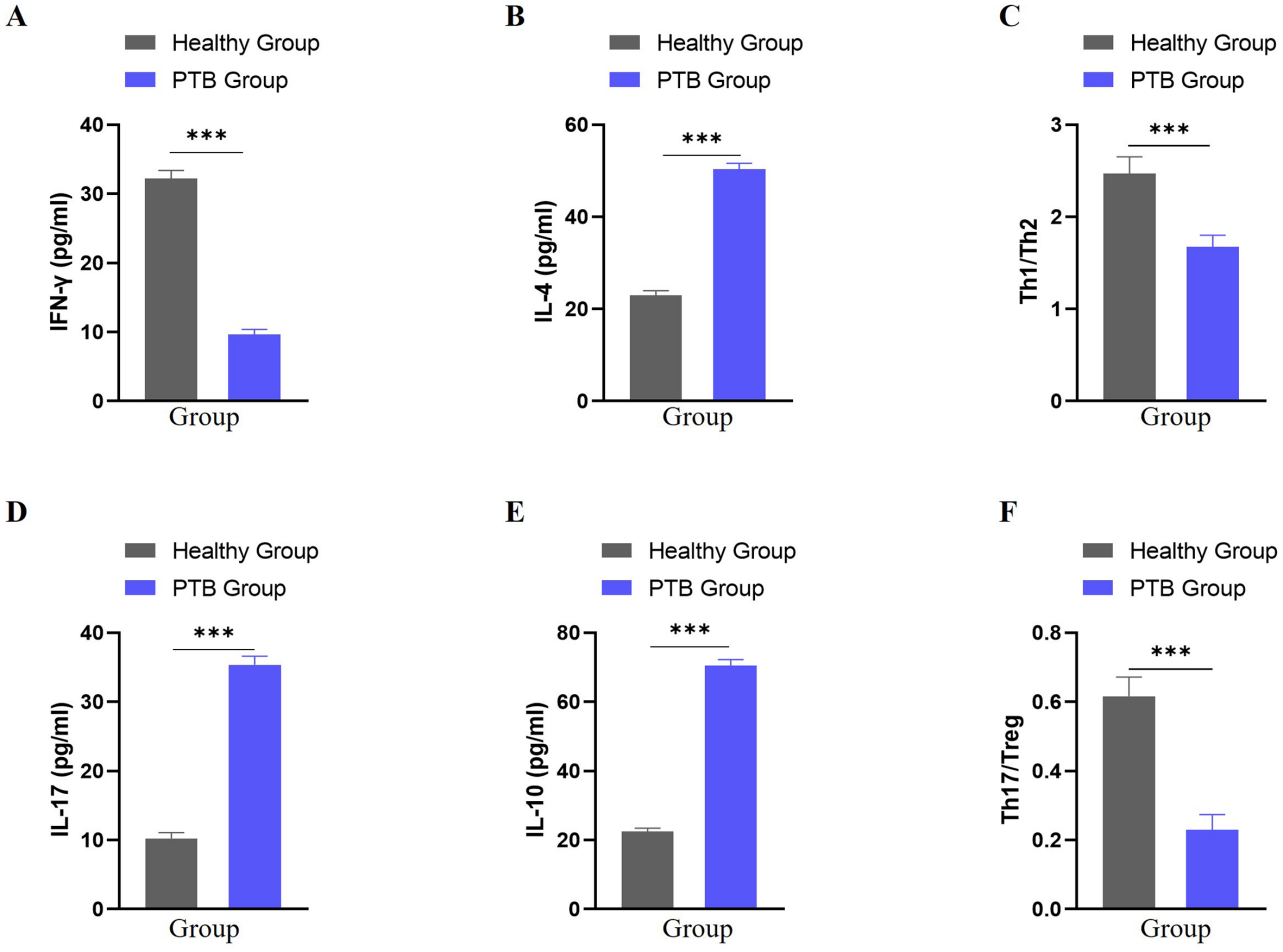
Comparison of Th1/Th2 and Th17/Treg cytokine levels between Healthy and PTB groups. **(A)** IFN-γ levels; **(B)** IL-4 levels; **(C)** Th1/Th2 ratio; **(D)** IL-17 levels; **(E)** IL-10 levels; **(F)** Th17/Treg ratio. Data are presented as mean ± SD. ***p < 0.001 vs. Healthy group.

**Figure 2:**
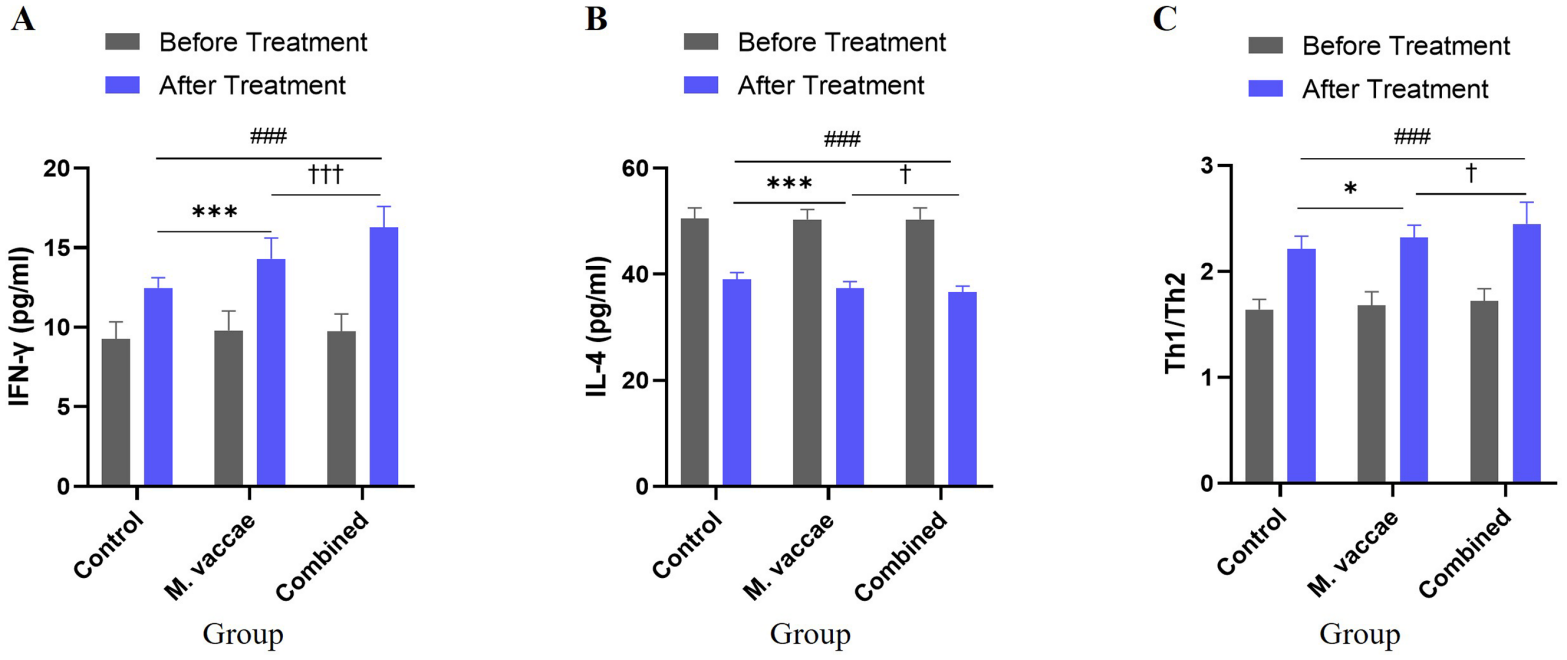
Changes in Th1/Th2 cytokine levels before and after 4 months of treatment in patients with initial smear-negative pulmonary tuberculosis. **(A)** IFN-γ; **(B)** IL-4; **(C)** Th1/Th2 ratio. Values are mean ± SD. *p < 0.05, ***p < 0.001 (Control vs. *M. vaccae*); ###*p* < 0.001 (Control vs. Combined); †*p* < 0.05, †††*p* < 0.001 (*M. vaccae* vs. Combined).

After 4 months of treatment, the Combined Therapy Group demonstrated superior clinical efficacy in patients with initial smear-negative pulmonary tuberculosis compared to the other groups (Table 7). This group achieved the highest total effective rate, significantly outperforming both the Control and *M. vaccae* groups (*p* < 0.001). The *M. vaccae* group showed intermediate improvement, whereas the Control Group exhibited the lowest overall efficacy (see Figures 1, 2).

**Table 7:** Efficacy analysis of patients with initial smear-negative pulmonary tuberculosis after 4 months of treatment.

Group	*n*	Cured, *n* (%)	Marked effect, *n* (%)	Effective, *n* (%)	Ineffective, *n* (%)	Total effective, *n* (%)
Control	134	9 (6.72)	32 (23.88)	61 (45.52)	32 (23.88)	103 (76.12)
*M. vaccae*	130	16 (12.31)	36 (27.69)	51 (39.23)	27 (20.77)	103 (79.23)
Combined Therapy	127	27 (21.26)^#†^	41 (32.26)^#†^	46 (36.22)^#†^	13 (10.24)^#†^	114 (89.76)^#†^

Overall differences among groups were assessed using the Kruskal–Wallis test (*p* = 0.013), followed by post hoc pairwise comparisons using the Mann–Whitney U test with Bonferroni correction, which showed significant differences between the Control and Combined Therapy groups (*p* = 0.013) and between the *M. vaccae* and Combined Therapy groups (*p* = 0.034), whereas no significant difference was found between the Control and *M. vaccae* groups (*p* = 0.258). ^#^*p* < 0.05 vs Control for Combined group; ^†^*p* < 0.05 vs *M. vaccae* for Combined group.

## 4 Discussion

Cell-mediated immunity plays a crucial role in the development and resolution of tuberculosis. *M. vaccae*, a rapidly growing non-tuberculous mycobacterium isolated from cow’s udders in 1964, is rich in antigens similar to those of *Mycobacterium tuberculosis* (21). It possesses immunomodulatory functions that promote the transformation, proliferation of T lymphocytes, and the release of various lymphokines, thereby inhibiting pathological damage caused by hypersensitivity reactions to infections such as tuberculosis (22).

Th1, Th2, Th17, and Treg are important CD4 + T cell subsets that participate in host defense, immune responses, and the pathological processes of infectious diseases by secreting a variety of cytokines (23). It is believed that the development of PTB is associated with the balance of Th1/Th2 and Th17/Treg, and the levels of related cytokines IFN-γ, IL-4, IL-10, and IL-17 are abnormally altered after *Mycobacterium tuberculosis* infection, with their levels closely correlating with the severity of PTB (24, 25). This study’s results show that the ratios of Th1/Th2 and Th17/Treg in patients with initial smear-negative PTB are significantly lower than in healthy individuals, and the levels of IFN-γ are markedly lower, while IL-4, IL-17, and IL-10 levels are significantly higher than in healthy individuals, consistent with previous research findings (26). Therefore, restoring the balance of Th1/Th2 and Th17/Treg, improving the secretion levels of related cytokines, and enhancing the immune function of PTB patients are of great significance for the prevention and treatment of PTB.

*Mycobacterium vaccae*, recommended as a bidirectional immune modulator in tuberculosis research and development strategies, significantly promotes T-cell transformation, proliferation, and the release of lymphokines, inhibiting hypersensitivity reactions caused by tuberculosis infection, and improving pathological damage (27). Initially used for the prevention of PTB, *M. vaccae* has gradually been used in the adjunctive treatment of PTB in recent years, but there are few reports on its efficacy. Studies have shown that adjunctive treatment with *M. vaccae* can effectively shorten the treatment period of smear-negative PTB (28), significantly improve the lesion absorption rate in patients with initial drug-sensitive PTB, and also show certain efficacy and safety in the retreatment of tuberculosis and multidrug-resistant tuberculosis, such as improving the sputum smear conversion rate, cavity closure rate, and lesion absorption rate in patients with retreatment smear-positive PTB and multidrug-resistant PTB, effectively improving patient immune function and reducing the one-year relapse rate (19, 29). However, the effect of *M. vaccae* on the immunity and efficacy of patients with initial smear-positive PTB is not clear at present. In our study, although *M. vaccae* alone significantly improved immune function (Th1/Th2 and Th17/Treg balance and cytokine levels), clinical and radiological improvements were not statistically significant compared with the control group. This may be due to its primary immunomodulatory role rather than direct lesion resolution, the relatively short treatment duration, and the limited sample size.

Reviewing existing research, it is found that combining *M. vaccae* treatment with conventional anti-tuberculosis treatment is more conducive to improving clinical efficacy. Some studies (30) have shown that compared with the combination of *M. vaccae* and conventional anti-tuberculosis treatment, the combination of conventional anti-tuberculosis treatment with *M. vaccae* and linezolid can significantly promote the healing of lesions in patients with multidrug-resistant tuberculosis, effectively improve the sputum bacteriuria conversion rate, and the comprehensive treatment effect is definite, while also effectively improving the level of T cell subset CD4 + and the CD4/CD8 ratio in patients, enhancing the patient’s immune function; studies have shown that using *M. vaccae* and Sha Shen Mai Dong Tang for the treatment of PTB on the basis of conventional anti-tuberculosis treatment has a definite curative effect, and compared with the conventional anti-tuberculosis treatment group, it can significantly improve the lesion absorption of patients, improve the sputum smear conversion rate at the end of 2 and 6 months, and reduce the incidence of adverse reactions to anti-tuberculosis drugs. However, there are still few studies on the use of *M. vaccae* in combination with other drugs for adjunctive treatment on the basis of conventional anti-tuberculosis treatment, and its safe and effective combination plan still needs further exploration.

Previous studies have reported inconsistent results regarding the efficacy of adjunctive vitamin D therapy in tuberculosis. A large individual participant data meta-analysis demonstrated that vitamin D supplementation did not significantly accelerate sputum culture conversion overall but showed beneficial effects in certain subgroups, such as patients with multidrug-resistant tuberculosis (31). These findings suggest that the therapeutic effect of vitamin D may depend on disease characteristics, host immune status, and treatment strategies. This study further demonstrates that the combination of *M. vaccae* and vitamin D adjunctive treatment significantly improved the balance of Th1/Th2 and Th17/Treg in peripheral blood, enhanced IFN-γ levels, and decreased IL-4, IL-17, and IL-10 levels compared with the control group and *M. vaccae* alone. Clinical outcomes, including sputum smear conversion and overall treatment efficacy after 6 months, were also superior to those in the *M. vaccae* group and control group. These findings suggest a synergistic effect between *M. vaccae* and vitamin D rather than the effect being solely attributable to vitamin D. However, since a vitamin D-only group was not included in this study, the independent contribution of vitamin D cannot be fully determined. Future studies should include a vitamin D monotherapy group to clarify its individual effect. Additionally, the potential influence of unmeasured comorbidities on study outcomes cannot be excluded. Although severe comorbid conditions were part of the exclusion criteria, other minor or undetected comorbidities may have affected immune responses or clinical outcomes. Future studies should collect and analyze detailed comorbidity data to better account for their potential confounding effects. The improvement in pulmonary lesions and cavity closure in the combined group was better than in the control group, but not significantly different from the *M. vaccae* group, possibly due to the small sample size and single-center design. Overall, conventional anti-tuberculosis treatment combined with *M. vaccae* and vitamin D is effective, enhancing CD4 + T cell subset balance, cytokine levels, and patient immunity, exhibiting a synergistic anti-tuberculosis effect.

## 5 Conclusion

In summary, the conventional anti-tuberculosis combined with *M. vaccae* and vitamin D adjunctive treatment can promote the recovery of the balance of CD4 + T cell subsets Th1/Th2 and Th17/Treg in patients, effectively improve the levels of cytokines IFN-γ, IL-4, IL-17, and IL-10, and the clinical efficacy is significant.

## Data Availability

The raw data supporting the conclusions of this article will be made available by the authors, without undue reservation.
